# LncRNA DLX6-AS1 promoted cancer cell proliferation and invasion by attenuating the endogenous function of miR-181b in pancreatic cancer

**DOI:** 10.1186/s12935-018-0643-7

**Published:** 2018-09-18

**Authors:** Yong An, Xue-min Chen, Yong Yang, Feng Mo, Yong Jiang, Dong-lin Sun, Hui-hua Cai

**Affiliations:** Department of Hepatobiliary Surgery, The First People’s Hospital of Changzhou, The Third Affiliated Hospital of Soochow University, 185 Juqian Street, Changzhou, 213000 Jiangsu China

**Keywords:** Pancreatic cancer, lncRNA DLX6-AS1, Proliferation, Migration and invasion, miR-181b, Zinc finger E-box-binding homeobox 2

## Abstract

**Background:**

Pancreatic cancer, one of the most aggressive malignancies, ranks the fourth cause of cancer-related death worldwide. Aberrantly expressed long non-coding RNAs (lncRNAs) functioned as oncogenes or tumor suppressors in pancreatic cancer. This study aimed to determine the expression of lncRNA DLX6 antisense RNA 1 (DLX6-AS1) in pancreatic cancer tissues and to explore the DLX6-AS1-related pathway in pancreatic cancer.

**Materials and methods:**

The gene expression levels were determined by quantitative real-time PCR, and protein expression levels were determined by western blot assay. CCK-8 assay, colony formation assay and Transwell migration and invasion assays were used to examine cell proliferation, migration and invasion. Luciferase reporter assay was used to confirm the binding between DLX6-AS1and its potential targets. In vivo study used the mouse xenograft model to test the anti-tumor effect of DLX6-AS1 knockdown.

**Results:**

The high expression of DLX6-AS1 was observed in pancreatic cancer tissues, and high expression of DLX6-AS1 was positively correlated with larger tumor size, advanced TNM stage and lymph node metastasis. Knockdown of DLX6-AS1 dramatically impaired cancer cell proliferation, migration and invasion. MiR-181b was the downstream target of DLX6-AS1. Knockdown of miR-181b reversed the suppression of cell viability, migration and invasion abilities caused by DLX6-AS1 knockdown. MiR-181b was found to target Zinc finger E-box-binding homeobox 2 and to modulate epithelial-mesenchymal transition. Furthermore, DLX6-AS1 knockdown inhibited tumor growth and tumor metastasis in vivo.

**Conclusion:**

Collectively, our data suggested that DLX6-AS1 promotes cancer cell proliferation and invasion by attenuating the endogenous function of miR-181b in pancreatic cancer.

## Background

Pancreatic cancer, one of the most aggressive malignancies, ranks the fourth cause of cancer-related death. Currently, there is lack of sensitive screening for early stage tumors. Up to date, surgery is the effective treatment for pancreatic cancer, however, the 5-year survival rate of the patients with pancreatic cancer is lower than 10% [1]. Unfortunately, 80% of the patients are not suitable for surgery and the recurrence rate in those who subjected to resection is very high [2]. Chemotherapy remains the primary treatment and the combination therapy including FOLFIRINOX, paclitaxel and gemcitabine is the first-line drug, but the outcome is not satisfactory [3, 4]. In terms of targeted drugs, the tyrosine-kinase inhibitor of epidermal growth factor receptor, Eroltinib, combined with gemcitabine, just made modest progress in survival benefit [5]. Consequently, the mechanisms underlying pancreatic cancer should be further investigated.

Recently, emerging evidence indicated the crucial roles of non-coding RNAs in various human diseases, and non-coding RNAs can be sub-grouped to small non-coding RNAs (< 200 nucleotides) and long non-coding RNAs (lncRNAs; > 200 nucleotides) [6]. LncRNAs take part in various physiological and pathological processes, including proliferation, differentiation, invasion, or chromosome inactivation [7]. Studies showed that lncRNA H19 was overexpressed in human pancreatic ductal adenocarcinoma and induced cancer cell proliferation by modulating cell cycle through E2F1 pathway [8]. LncRNA HOXA-AS2 was also up-regulated in pancreatic cancer tissues. Mechanistically, it promoted pancreatic cancer cell proliferation by interacting with enhancer of zeste homolog 2 and lysine specific demethylase 1 [9]. In addition, the microRNAs belongs to a class of small non-coding RNA with 18–22 nucleotides in length [10, 11]. Up to date, hundreds of identified miRNAs have been shown to function as key regulators in tumor development including pancreatic cancer. For example, miR-1271 served as a potent tumor suppressor by reducing AKT/mTOR signaling and promoting apoptosis [12]. Overexpression of miR-126 and miR-34a had superior anti-tumor effect in pancreatic cancer [13]. The lncRNA DLX6 antisense RNA 1 (DLX6-AS1) is a developmentally-regulated long non-coding RNA. According to GTEx Analysis, it is overexpressed in brain tissues in normal. High DLX6-AS1 expression was noticed in lung adenocarcinoma and associated with histological differentiation and TNM stage [14]. DLX6-AS1 was also up-regulated in hepatocellular carcinoma tissue and correlated with clinical prognosis [15]. However, how DLX6-AS1 regulates pancreatic cancer tumorigenesis and its underlying molecular mechanisms regarding pancreatic cancer development remain unknown.

In the present study, we detected the expression of DLX6-AS1 in pancreatic cancer tissues and cells and predicted its potentially targeted miRNA. We demonstrated that DLX6-AS1 up-regulated the expression of Zinc finger E-box-binding homeobox 2 (ZEB2) by sponging miR-181b, therefore promoting cell proliferation, invasion as well as epithelial-mesenchymal transition in pancreatic cancer cells.

## Materials and methods

### Human specimens

Eighty-four pairs of cancerous and adjacent normal tissues were collected from patients diagnosed with pancreatic cancer from 2015 to 2017 at the First People’s Hospital of Changzhou. All the specimens were frozen in liquid nitrogen and stored at − 80 °C for further use. The study was approved by the Ethics Committee of the First People’s Hospital of Changzhou and written informed consent was signed and returned by each patient before using the tissues and clinical data.

### Cell culture

Human pancreatic duct epithelial cell Line (HPDE6-C7), and human pancreatic cancer cell lines CAPAN-1, BxPC-3, SW 1990 and PANC-1were purchased from ATCC (Manassas, USA). CAPAN-1 and BxPC-3 cells were cultured in RPMI-1640 medium (Gibco, Thermo Fisher Scientific, Waltham, USA) supplemented with 10% fetal bovine serum (FBS, Gibco, Thermo Fisher Scientific). PANC-1cells were cultured in Dulbecco’s modified Eagle’s medium (Gibco, Thermo Fisher Scientific) supplemented with 10% FBS (Gibco Thermo Fisher Scientific). SW 1990 cells were cultured in Leibovitz’s L-15 medium and HPDE6-C7 was maintained in Keratinocyte Serum Free Medium supplemented with 25 mg/500 ml Bovine Pituitary Extract and 2.5 µg/500 ml Epidermal Growth Factor (Gibco, Thermo Fisher Scientific). They were all kept at 37 °C in a humidified atmosphere containing 5% CO_2_.

### Oligonucleotides and transfection

The small interfering RNAs (siRNAs) for DLX6-AS1 [siDLX6-AS1 and siDLX6-AS1(a)] and the respective negative control siRNA, miR-181b mimics (5′-AACAUUCAUUGCUGUCGGUGGGU-3′), miR-181b inhibitors (5′-ACCCACCGACAGCAAUGAAUGUU-3′) and the respective negative control miRNAs were purchased from RiboBio (Guangzhou, China). Cell transfections (final concentration for siRNA transfection: 100 nM, final concentration for miRNAs transfection: 50 nM) were performed using Lipofectamine 2000 (Invitrogen, Carlsbad, USA) according to the manufacturer’s protocol and the transfection efficiency was confirmed using quantitative real-time PCR (qRT-PCR). All the experiments were performed in triplicate.

### RNA isolation and qRT-PCR

Total RNA was extracted from tissue or cells through TRIzol reagent (Invitrogen, Carlsbad, USA). The extracted RNA was then reversely transcribed into cDNA using PrimeScript™ 1st strand cDNA Synthesis Kit (Takara, Dalian, China). Real-time PCR was performed with SYBR Premix Ex Taq (Takara, Dalian, China) in the ABI7500 system. GAPDH or U6 was used as internal control for data analysis. All the experiments were performed in triplicate.

### CCK-8 assay

For proliferation assay, the transfected cells were seeded in 96-well plates at the density of 5000 cells/well over night. At 0, 24, 48 and 72 h after transfection, the cells were incubated with 10 µl CCK-8 reagent for 2 h at 37 °C in the dark, and the OD value at 450 nm was read using a microplate reader (Bio-Rad Laboratories, Hercules, CA, USA) to determine the viability. All the experiments were performed in triplicate.

### Colony formation assay

The transfected cells were seeded in a 6-well plate at the density of 1000 cells/well and incubated in full culture medium at 37 °C. At 14 day, the cells were washed with PBS, fixed with methanol and stained with 1% crystal violet. The number of colonies was counted under a microscope. All the experiments were performed in triplicate.

### Transwell migration and invasion assays

Transwell migration and invasion assays were measured by Transwell chamber (8 µm pore size, Corning) without Matrigel for migration assay and with Matrigel for invasion assay. At 48 h after transfection, 1 × 10^5^ cells were cultured in the upper chamber with serum-free median. The lower chamber was filled with median containing 10% FBS as the chemoattractant. After incubation for 48 h, cells in the upper membrane were removed with cotton swab while those that migrated or invaded were fixed in methanol, stained with 0.1% crystal violet and quantified under a microscope. All the experiments were performed in triplicate.

### Luciferase reporter assays

DLX6-AS1 fragments with the wild type (WT) or mutant (MUT) miR-181b binding sites were cloned to generate the plasmids pmirGLO-DLX6-AS1-WT or pmirGLO- DLX6-AS1-MUT (Promega, Madison, USA). The 3′UTR of ZEB2 containing the wild type or mutant miR-181b binding sites were used to generate the plasmids pmirGLO-ZEB2-WT and pmirGLO-ZEB2-MUT (Promega). HEK293T cells were co-transfected with luciferase plasmids and miR-181b mimics, or mimics NC by using Lipofectamine 2000 reagent according to the manufacture’s instruction (Invitrogen). At 48 h after transfection, firefly and Renilla luciferase activities were measured by a Dual-Luciferase Reporter Assay System (Promega) and the experiments were performed in triplicate.

### Western blot

The tissue or cells were collected and total proteins were extracted and quantified. Equal amount of proteins was subjected to 10% SDS-PAGE and then transferred onto PVDF membranes (Millipore, Bedford, USA). After blocking with 5% fat-free milk, the membranes were probed with primary anti-ZEB2, anti-E-cadherin, anti-vimentin or anti-N-cadherin antibodies (Abcam, Cambridge, USA) at 4  °C overnight. Then membranes were incubated with horseradish peroxidase-conjugated IgG secondary antibody (Santa Cruz Biotechnology, Santa Cruz, USA) at 37  °C for 2 h. Enhanced chemiluminescence kit (Pierce, Waltham, China) with imaging system (Bio-Rad, Hercules, USA) were used to analyze the protein signals and β-actin was used as the internal control.

### Tumor growth and tumor metastasis assay in nude mice

Lentivirus expressing shDLX6-AS1 or shRNA control were designed and packaged by Genechem (Shanghai, China), and stable cell lines were established by infecting lentivirus into SW1990 cells and selected by puromycin (Sigma, St. Louis, USA). All the animal experiments were approved by the Animal Experimentation Ethics Committee of the First People’s Hospital of Changzhou Hospital. For the tumor growth assay, twelve female BABL/c athymic nude mice (4–5 weeks old) were subjected to flank subcutaneous injection of SW1990 cells stably expressing shDLX6-AS1 or shNC. Tumor volume was measured every 5 day from the 10 day to the 35 day after inoculation. The volume was calculated as: volume  = (length  ×  width^2^)/2. At 35 day, the mice were sacrificed and the tumors were removed, weighed and snap-frozen for RNA extraction. For the tumor metastasis assay, twelve female BABL/c athymic nude mice (4–5 weeks old) were subjected to tail vein injection of SW1990 cells stably expressing shDLX6-AS1 or shNC. At 35 day, all the mice were sacrificed and the lung were excised, and the number of metastatic nodules in the lung was counted.

### Statistical analysis

Data were presented as mean  ±  standard deviation. The data analysis was performed by using GraphPad Prism software (Version 5.0, GraphPad, San Diego, USA). The significant differences in the clinical parameters between low expression and high expression of DLX6-AS1 groups were analyzed by Chi square test. Significant differences for the mean values between groups were determined by the Student’s *t* test or one-way ANOVA, as appropriate. P value < 0.05 was considered statistically significant.

## Results

### Up-regulation of lncRNA DLX6-AS1 in pancreatic cancer tissues

**Fig. 1:**
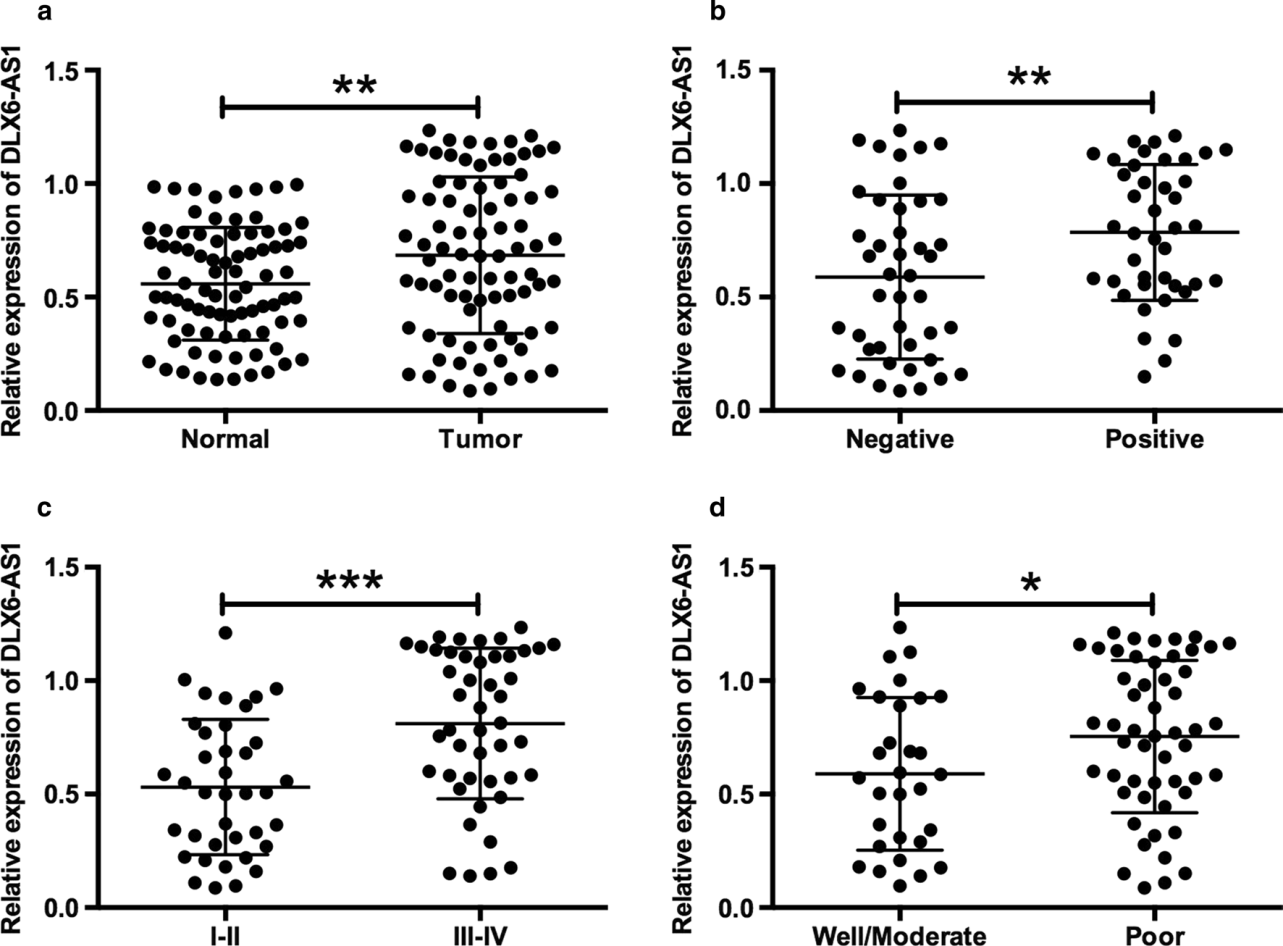
Up-regulation of lncRNA DLX6-AS1 in pancreatic cancer tissues. **a** The expression of DLX6-AS1 in pancreatic cancer tissues (n = 84) and normal adjacent pancreatic tissues (n = 84) was determined by qRT-PCR. **b** The expression of DLX6-AS1 in pancreatic cancer tissues from patients without lymph node metastasis (n = 43) and with lymph node metastasis (n = 41). **c** The expression of DLX6-AS1 in pancreatic cancer tissues from patients with TNM stages (I–II, n = 38; III-IV, n = 46). **d** The expression of DLX6-AS1 in well/moderate (n = 33) and poor differentiated (n = 51) pancreatic cancer tissues. *P < 0.05, **P < 0.01 and ***P < 0.001

The expression levels of lncRNA DLX6-AS1 in 84 pancreatic cancerous tissues and adjacent normal tissues were firstly evaluated by qRT-PCR. Expression level of DLX6-AS1 was up-regulated in cancerous tissues comparing with the adjacent normal counterparts (Fig. 1a). The expression of DLX6-AS1 in pancreatic cancer tissues was further divided into low expression group and high expression group based on the median values. High expression of DLX6-AS1 was positively correlated with larger tumor size, advanced TNM stage and lymph node metastasis (Table 1). Meanwhile, we have sub-grouped pancreatic cancerous tissues as lymph node metastasis negative and positive groups, low TNM stages (I-II) and high TNM stages (III-IV) groups, as well as well/moderate and poor differentiated groups. As shown in Fig. 1b–d, the expression level of DLX6-AS1 in the lymph node metastasis negative group, low TNM stages group or well/moderate group was lower than that in the lymph node metastasis positive, high TNM stages or poor differentiated groups.

**Table 1:** Correlation between lncRNA DLX6-AS1 expression and clinicopathological parameters of patients with pancreatic cancer

Clinical parameters	DLX6-AS1 expression		P value
	Low (n = 41)	High (n = 43)	
Gender			
Male	22	20	0.6627
Female	19	23	
Age (years)			
< 60	15	17	0.825
≥ 60	26	26	
Tumor size (cm)			
< 2	27	17	0.0181
≥ 2	14	26	
TNM stage			
I–II	25	13	0.0081
III–IV	16	30	
Tumor differentiation			
Well/Moderate	21	12	0.0438
Poor	20	31	
Lymph node metastasis			
Negative	28	15	0.0026
Positive	13	28	
Distant metastasis			
Negative	22	18	0.3822
Positive	19	25	

### Knockdown of lncRNA DLX6-AS1 suppressed pancreatic cancer cell proliferation, migration and invasion

**Fig. 2:**
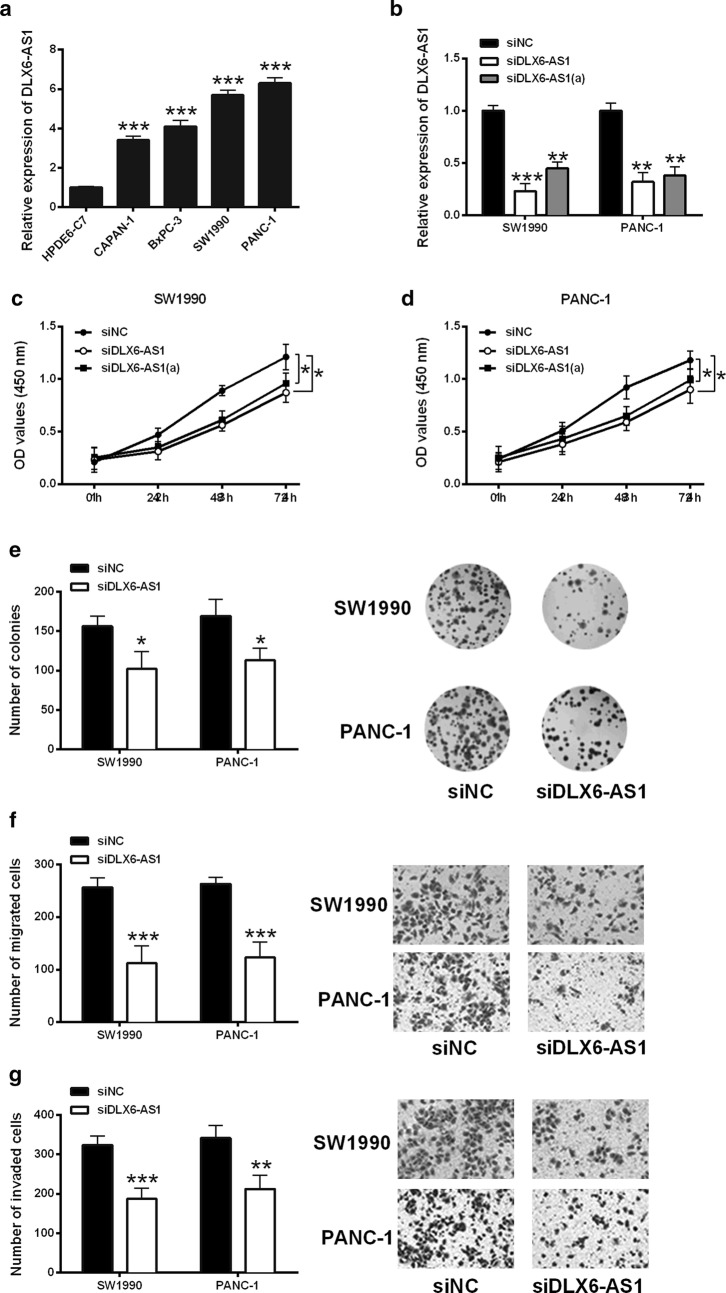
Knockdown of lncRNA DLX6-AS1 suppressed pancreatic cancer cell proliferation, migration and invasion. **a** The expression of DLX6-AS1 in human pancreatic cancer cell lines and human pancreatic duct epithelial cell line (HPDE6-C7) was determined by qRT-PCR. **b** DLX6-AS1 siRNAs [siDLX6-AS1 and siDLX6-AS1(a)] transfection suppressed the expression of DLX6-AS1 in SW1990 cell sand PANC-1 cells. The cell proliferation of **c** SW1990 cells and **d** PANC-1 cells transfected with siNC, siDLX6-AS1 or siDLX6-AS1(a) was determined by CCK-8 assay. **e** Colony formation assay was performed to analyze the cell growth of SW1990 and PANC-1 cells transfected with siNC or siDLX6-AS1. **f** Cell migration and **g** cell invasion of SW1990 and PANC-1 cells transfected with siNC or siDLX6-AS1 were determined by Transwell migration assay and Transwell invasion assay, respectively. *P < 0.05, **P < 0.01 and ***P < 0.001

The expression of DLX6-AS1 in human pancreatic cancer cell lines (CAPAN-1, BxPC-3, SW1990 and PANC-1) and human pancreatic duct epithelial cell line (HPDE6-C7) was determined by qRT-PCR. Elevated expression of DLX6-AS1 was observed in pancreatic cancer cell lines compared with HPDE6-C7 cells (Fig. 2a). To investigate the biological function of DLX6-AS1 in pancreatic cancer cells, CCK-8 assay, colony formation assay and Transwell migration/invasion assay were performed to measure the cell proliferation, migration and invasion in SW1990 and PANC-1 cells transfected with DLX6-AS1 siRNAs or scrambled siRNA. The knockdown efficiency of DLX6-AS1 siRNAs [si-DLX6-AS1 and DLX6-AS1(a)] was firstly confirmed in both cell lines (Fig. 2b). CCK-8 assay results showed that both siDLX6-AS1 and siDLX6-AS1(a) suppressed cell viability of pancreatic cancer cells in a time-dependent number (Fig. 2c, d). As both siRNAs were effective in suppressing DLX6-AS1 expression and cell viability, siDLX6-AS1 was used for further experimentation. Colony formation assay results demonstrated that DLX6-AS1 knockdown reduced the number of colonies compared to siNC group (Fig. 2e). Additionally, migration/invasion assay results showed that siDLX6-AS1 transfection suppressed cell migration and invasion abilities of SW1990 and PANC-1cells (Fig. 2f, g).

### DLX6-AS1 targeted miR-181b in pancreatic cancer cells

**Fig. 3:**
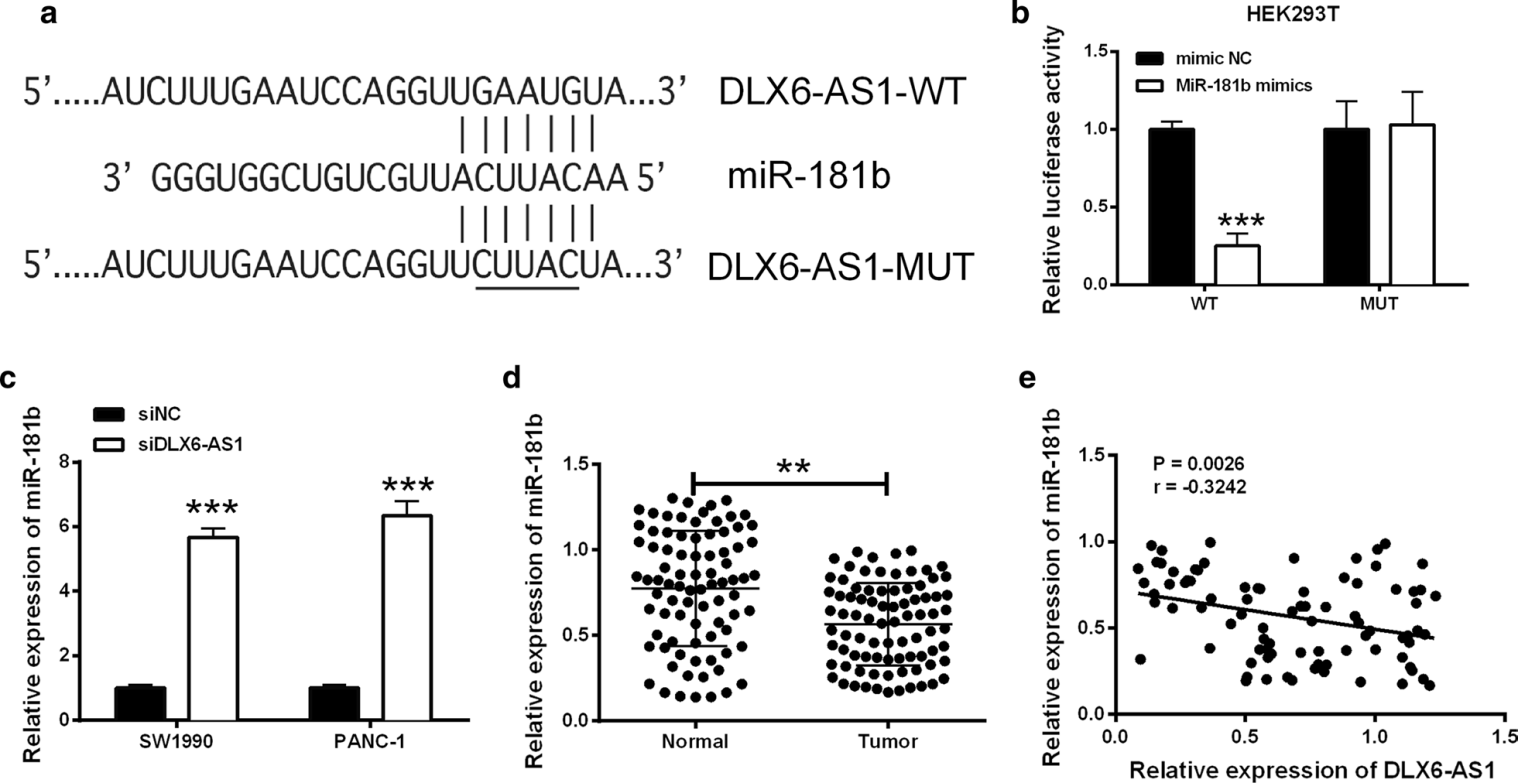
LncRNA DLX6-AS1 negatively regulated the expression of miR-181b. **a** The putative binding sites between DLX6-AS1 and miR-181b. **b** Luciferase activity was determined in HEK293T cells co-transfected with miRNAs (mimics NC or miR-181b mimics) and reporter vector containing DLX6-AS1 segments (WT or MUT) binding to miR-181b. **c** The expression of miR-181b in SW1990 and PANC-1 cells transfected with siNC or siDLX6-AS1 was determined by qRT-PCR. **d** The expression of miR-181b was down-regulated in pancreatic cancer tissues (n = 84) compared to normal adjacent pancreatic cancer tissues (n = 84). **e** The correlation between miR-181b expression and DLX6-AS1 expression in pancreatic cancer tissues was analyzed by Spearman’s correlation test (r = − 0.3242, P = 0.0026). **P < 0.01 and ***P < 0.001

We used the DIANA tools to predict the potential targets of DLX6-AS1 and found that miR-181b contain the complementary binding site of DLX6-AS1. To confirm this, luciferase reporter vectors containing the wild type or mutated binding sites were constructed. They were co-transfected with miR-181b mimics or mimic NC into HEK293T cells. The results showed that miR-181b reduced the luciferase activity of wild type DLX6-AS1, but had no effect on the mutant one (Fig. 3a, b). Furthermore, SW1990 and PANC-1cells were transfected with siDLX6-AS1or siNC. The qRT-PCR results revealed that silencing DLX6-AS1 increased the expression level of miR-181b in both cell lines (Fig. 3c). Meanwhile, the miR-181b expression level in pancreatic cancer tissues was lower than that in the adjacent normal tissues (Fig. 3d). Furthermore, a negative correlation between DLX6-AS1 and miR-181b expression in the pancreatic cancer tissues was observed (Fig. 3e).

### DLX6-AS1 regulated cell viability and invasion through miR-181b

**Fig. 4:**
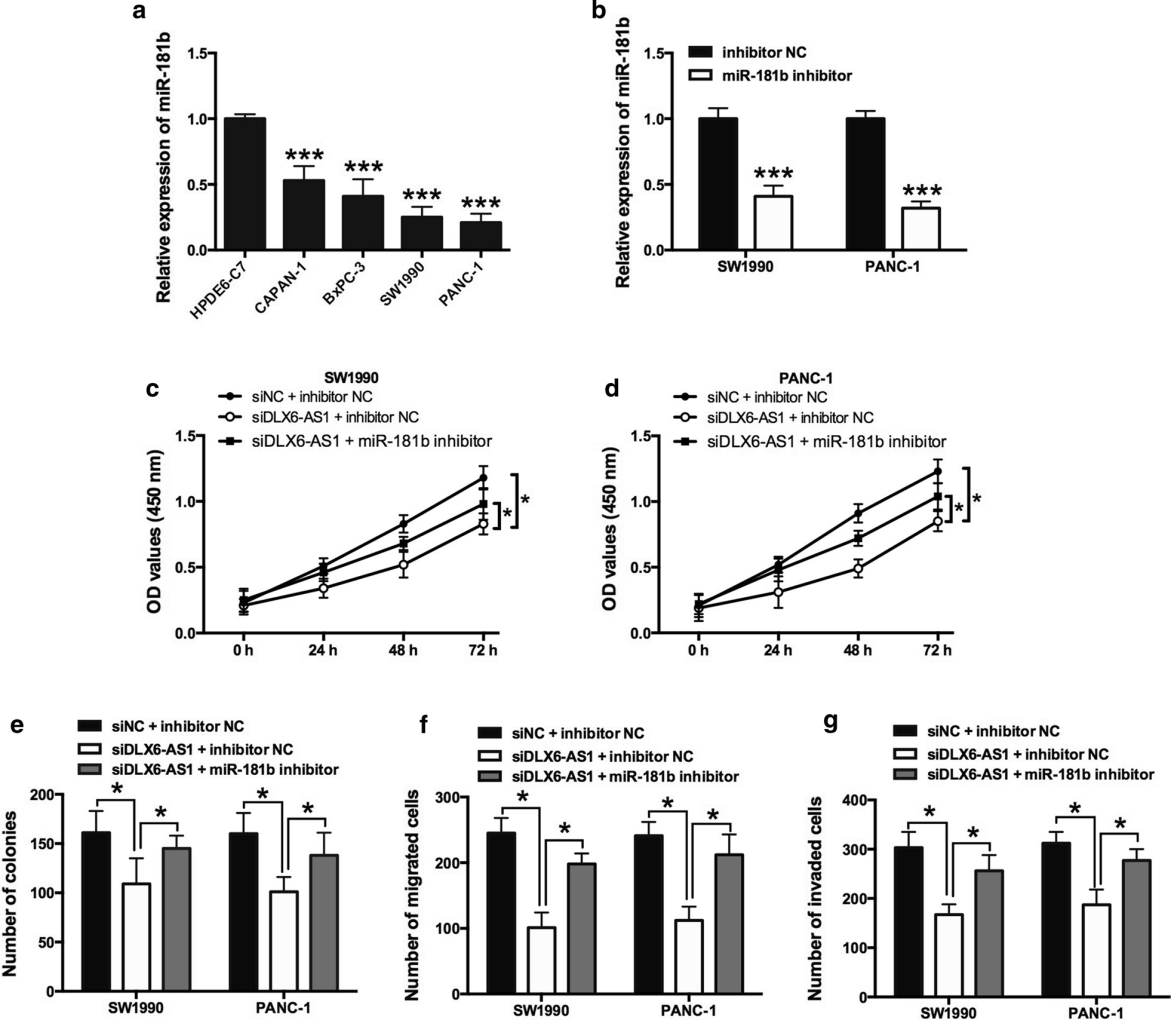
Knockdown of miR-181b attenuated DLX6-AS1-mediated effects on pancreatic cancer cell proliferation, migration and invasion. **a** The expression of miR-181b in human pancreatic cancer cell lines and human pancreatic duct epithelial cell line (HPDE6-C7) was determined by qRT-PCR. **b** The expression of miR-181b in SW1990 and PANC-1 cells transfected inhibitor NC or miR-181b inhibitor was determined by qRT-PCR. The cell proliferation of **c** SW1990 and **d** PANC-1 cells co-transfected with siNC + inhibitor NC, siDLX6-AS1 + inhibitor NC, or siDLX6-AS1 + miR-181b inhibitor was determined by CCK-8 assay. **e** Cell growth, **f** cell migration and **g** cell invasion of SW1990 and PANC-1 cells co-transfected with siNC + inhibitor NC, siDLX6-AS1 + inhibitor NC, or siDLX6-AS1 + miR-181b inhibitor were determined by colony formation assay, Transwell migration assay and Transwell invasion assay, respectively. *P < 0.05 and ***P < 0.001

Firstly, we have checked the expression of miR-181b in four human pancreatic cancer cell lines and one normal pancreatic duct epithelial cell line (HPDE6-C7). Decreased expression of miR-181b was observed in cancer cell lines compared with HPDE6-C7 cells and miR-181b inhibitor transfection reduced the expression of miR-181b in both SW1990 and PANC-1 cells (Fig. 4a, b). Previously, we have shown that knockdown of DLX6-AS1 suppressed pancreatic cancer cell proliferation, migration and invasion. To explore whether DLX6-AS1 exerted biological effects through miR-181b, the cells were treated with siDLX6-AS1, together with miR-181b inhibitor or inhibitor NC. CCK-8 assay showed that miR-181b inhibitor reversed the reduction of cell viability as well as colony formation ability caused by DLX6-AS1 knockdown in SW1990 and PANC-1 cells (Fig. 4c–e). In addition, cell migration and invasion assay showed that miR-181b inhibitor abrogated the inhibitory effect of DLX6-AS1 knockdown on cell migration and invasion in SW1990 and PANC-1 cells (Fig. 4f, g).

### miR-181b targeted ZEB2 and regulated epithelial-mesenchymal transition (EMT) in pancreatic cancer cells

**Fig. 5:**
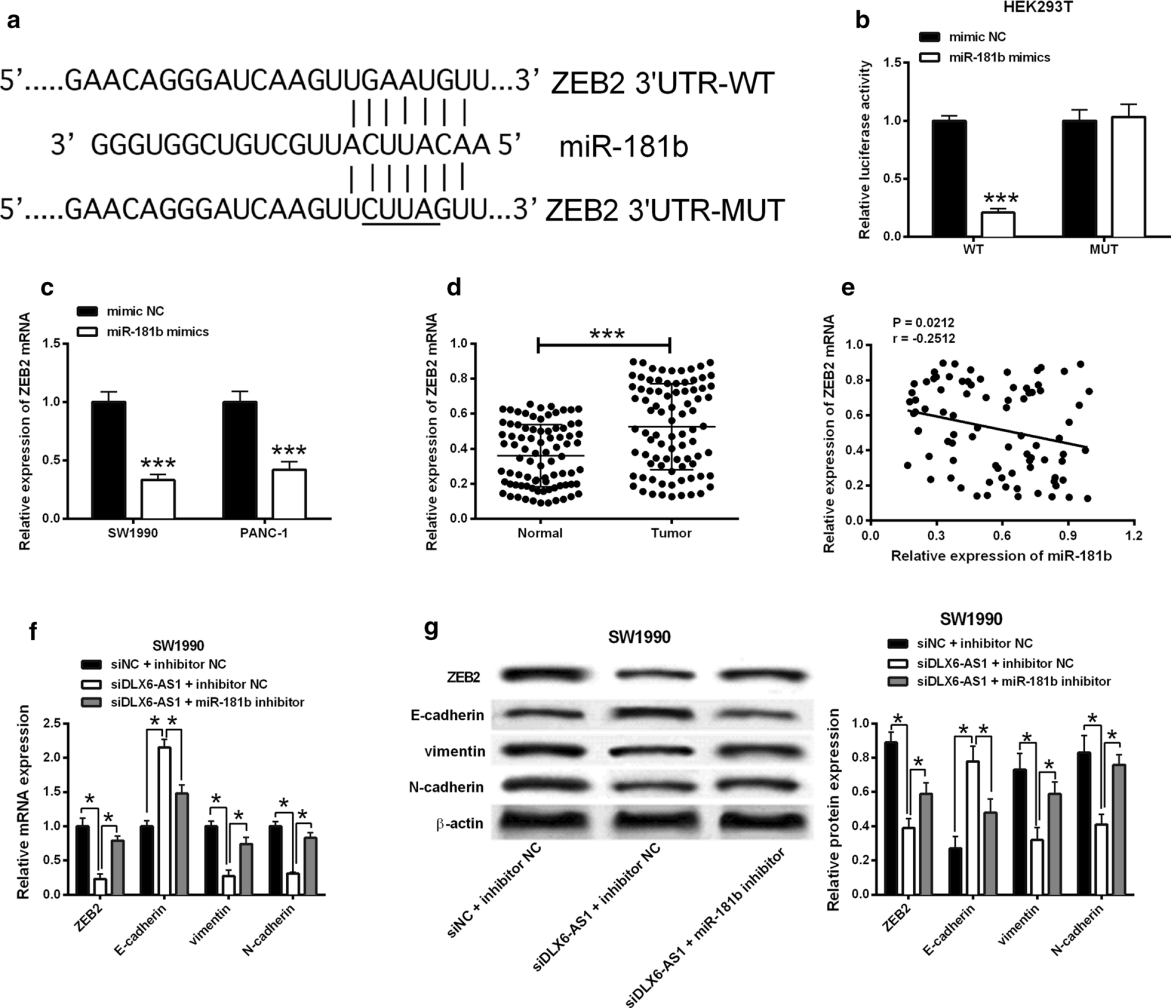
ZEB2 is a downstream target of miR-181b. **a** The putative binding sites between miR-181b and ZEB2 3′UTR. **b** Luciferase activity was determined in HEK293T cells co-transfected with miRNAs (mimics NC or miR-181b mimics) and reporter vector containing ZEB2 3′UTR segments (WT or MUT) miR-181b. **c** The mRNA expression of ZBE2 in SW1990 and PANC-1 cells transfected with mimic NC or miR-181b mimc was determined by qRT-PCR. **d** The mRNA expression of ZEB2 was up-regulated in pancreatic cancer tissues (n = 84) compared to normal adjacent pancreatic cancer tissues (n = 84). **e** The correlation between miR-181b expression and ZEB2 mRNA expression in pancreatic cancer tissues was analyzed by Spearman’s correlation test (r = − 0.2512, P = 0.0212). **f** The mRNA and **g** protein expression of ZEB2, E-cadherin, vimentin and N-cadherin in SW1990 cells co-transfected with siNC + inhibitor NC, siDLX6-AS1 + inhibitor NC or siDLX6-AS1 + miR-181b inhibitor. *P < 0.05 and ***P < 0.001

Similarly, we used the TargetScan to predict the potential targets of miR-181b and found that ZEB2 was one of the targets of miR-181b. Luciferase reporter assay showed miR-181b mimics reduced the luciferase activity of wild type ZEB2 3′UTR, but had no effect on the mutant one (Fig. 5a, b). Next, the mRNA expression level of ZEB2 was measured in cell lines and patient tissues. ZEB2 expression was reduced by miR-181b mimics transfection in SW1990 and PANC-1 cells (Fig. 5c) and the mRNA expression level of ZEB2 was higher in pancreatic tumor tissues than that in the paired adjacent ones (Fig. 5d). In addition, a negative correlation between miR-181b and ZEB2 mRNA expression in pancreatic cancer tissues was observed (Fig. 5c–e). Furthermore, the mRNA and protein levels of ZEB2 and EMT markers including E-cadherin, vimentin and N-cadherin were examined. DLX6-AS1 knockdown decreased the mRNA and protein expression levels of ZEB2, vimentin and N-cadherin, and increased the mRNA and protein expression levels of E-cadherin, which was abrogated by miR-181b inhibitor transfection (Fig. 5f, g).

### DLX6-AS1 knock-down inhibited tumor growth in vivo

**Fig. 6:**
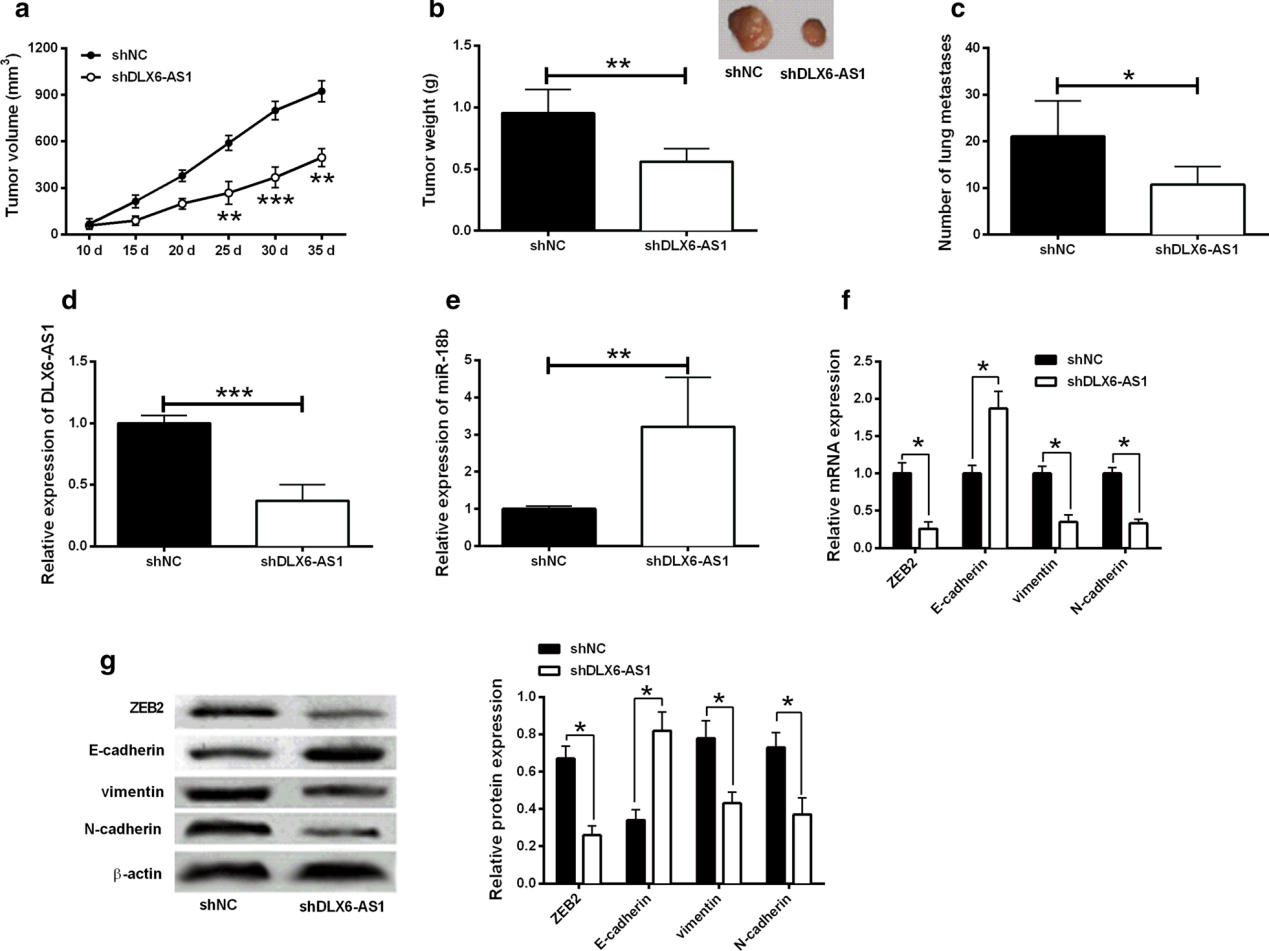
Knockdown of DLX6-AS1 inhibited in vivo tumor growth in nude mice. **a** The tumor volume at 25, 30 and 35 days following tumor cells injection was significantly lower in shDLX6-AS1 group than that in shNC group. **b** The weight of dissected tumors in shDLX6-AS1 group was lower than that in shNC group. **c** The number of metastatic nodules in the lung from shDLX6-AS1 group was reduced compared to that in shNC group. The expression of **d** DLX6-AS1 and **e** miR-181b in dissected tumor tissues from shNC group and shDLX6-AS1 group were determined qRT-PCR. **f** The mRNA and **g** protein expression of ZEB2, E-cadherin, vimentin and N-cadherin in the dissected tissues were determined by qRT-PCR and western blot, respectively. *P < 0.05, **P < 0.01 and ***P < 0.001

SW1990 cells stably transfected with shDLX6-AS1 or shNC were injected into the nude mice. As shown in Fig. 6a, DLX6-AS1 knockdown inhibited tumor growth comparing with shNC group. The tumor volume at 25, 30 and 35 d following injection was significantly lower in shDLX6-AS1 group than that in shNC group. The weight of dissected tumors in shDLX6-AS1 group was also lower than that in shNC group (Fig. 6a, b). The number of metastatic nodules in the lung from the shDLX6-AS1 group was significantly reduced compared to shNC group (Fig. 6c). Moreover, in consistent with the in vitro results, the expression of DLX6-AS1 was decreased and the expression of miR-181b was increased in shDLX6-AS1 group (Fig. 6d, e). In addition, the mRNA and protein levels of ZEB2, vimentin and N-cadherin was down-regulated while E-cadherin was up-regulated in shDLX6-AS1 (Fig. 6f, g).

## Discussion

In the early stage of pancreatic cancer, there are almost no symptoms. When patients have typical symptoms, they have already reached an advanced stage and showed tumor metastasis [16]. As a result, the prognosis for pancreatic cancer is poor and the 5-year overall survival is very low with median survival of only 6 months [17, 18]. These make us to seek more effective therapies and advanced strategies for the treatment of pancreatic cancer.

Accumulating evidence has demonstrated the critical roles of lncRNAs in regulating pathological processes of pancreatic cancer. For instance, ectopic expression of AB209630 suppressed cell proliferation ability in pancreatic ductal adenocarcinoma cells resistant to gemcitabine through blocking the PI3K/AKT signaling pathway [19]. UCA1 was up-regulated in pancreatic cancer tissues and correlated with poor clinical outcome, and promoted cell migration and invasion by the Hippo signaling pathway [20]. XLOC_000647 served as a tumor suppressor, impairing cell proliferation, invasion, and epithelial-mesenchymal transition abilities through NLRP3 inhibition in pancreatic cancer [21]. In this study, we identified the upregulation of DLX6-AS1 in pancreatic cancer. Besides, knockdown of DLX6-AS1 dramatically impaired pancreatic cancer cell proliferation, migration and invasion.

Recently, lncRNAs was found to function as competing endogenous RNAs to regulate the expression level of miRNA, thus playing crucial role in the development of cancer. For better understanding the mechanisms of DLX6-AS1 in pancreatic cancer, we found that miR-181b was of the downstream targets, and miR-181b has been reported as tumor suppressor in various cancers. In glioblastoma, lower level of miR-181b was correlated with more advanced grade patients. MiR-181b can modulate vascular cell adhesion molecule 1 expression and monocyte adhesion in an epidermal growth factor receptor-dependent pathway [22]. MiR-181b suppressed cell cycle, cell viability and proliferation through down-regulation of FAMLF in burkitt lymphoma cells [23]. In breast cancer, ectopic expression of miR-181b inhibited the increase of NF-κB level induced by chemokine ligand 18 in cancer cells, thus suppressing cell survival rate and migration [24]. Consistent with the above reports, our results showed that miR-181b functioned as a tumor suppressor in pancreatic cancer. Its inhibition reversed the reduction of cell viability, migration and invasion abilities caused by DLX6-AS1 knockdown.

Furthermore, we investigated the molecular basis of miR-181b through TargetScan and found that ZEB2 was the downstream target of miR-181b. Here we found that the expression level of ZEB2 in pancreatic cancer tissues was higher than that in the paired adjacent ones. ZEB2 belongs to the ZEB proteins family, and was reported to interact with SMAD protein, involving TGF-β-induced EMT. During EMT, epithelial cells obtain the motile and invasive abilities typical of mesenchymal cells, which is an important process for cancer metastasis and associated with poor prognosis [25, 26]. In terms of molecular level, this process is indicated by the expression changes of some markers, including loss of epithelial markers and the gain of mesenchymal markers. ZEB2 is reported to be a repressor of E-cadherin and the cadherin switching, like decrease in E-cadherin and increase in N-cadherin is a feature of EMT in malignant tumors [27, 28]. In the present study, in vitro data showed that DLX6-AS1 knockdown decreased ZEB2 expression level, followed by the increase of E-cadherin and decrease of vimentin and N-cadherin, which was abrogated by miR-181b inhibitors. In the mouse xenograft models, the mRNA level of ZEB2, vimentin and N-cadherin was downregulated while E-cadherin was up-regulated in the DLX6-AS1 knock-down group with reduced tumor size and suppressed tumor metastasis. Taken together, the effect of DLX6-AS1 on EMT depends on the regulation of ZEB2 through targeting miR-181b. In addition, our results showed that the effects of DLX6-AS1 knockdown on survival were marginal, and the effects of DLX6-AS1 knockdown and the rescue experiments have a greater effect on cell migration and EMT-related markers expression, indicating that dysregulation of DLX6-AS1 may be more important for pancreatic cancer metastasis rather than tumor maintenance.

## Conclusion

In conclusion, we investigated the uncharacterized role of DLX6-AS1 in pancreatic cancer and demonstrated that DLX6-AS1 promoted cancer cell proliferation and invasion by attenuating the endogenous function of miR-181b.
